# Healthspan-Lifespan Trade-off Under Accelerated Biological Aging: Evidence From The Health And Retirement Study

**DOI:** 10.1093/geroni/igaf122.3600

**Published:** 2025-12-31

**Authors:** Heming Pei, Arun Balachandran, Yifan Shi, Daniel Belsky

**Affiliations:** UNC Gillings School of Global Public Health University of North Carolina at Chapel Hill, Chapel Hill, North Carolina, United States; Columbia University, New York City, New York, United States; New York University, New York, New York, United States; Columbia University, New York, New York, United States

## Abstract

While life expectancy (LE) has increased globally, the rise in healthy life expectancy (HLE) has not kept pace. This widening gap between HLE and LE—years lived in poor health—could be impacted by the pace of physiological deterioration. This study investigates how accelerated biological aging affects transitions between health states and to assess how it contributes to the compression or expansion of healthspan relative to lifespan, and whether this effect is more pronounced in socially disadvantaged groups. Using longitudinal data from the U.S. Health and Retirement Study, we quantified biological aging with the Pace of Aging metric. Health transitions—including chronic disease, functional decline, cognitive impairment, and death—were modeled using a continuous-time Markov multi-state approach to simulate and calculate healthspan and lifespan across different aging profiles. The analysis revealed that individuals with faster pace of aging had increased risk of transitioning from healthy to unhealthy states, more so than the risk of death once already unhealthy. This imbalance led to an extended duration of life spent in poor health. The HLE–LE gap was consistently wider among those aging faster across all outcomes. And this effect was strongest in individuals with lower educational attainment, minority status, or unhealthy behaviors, suggesting systemic inequities amplify the impact of biological aging. Accelerated biological aging predicts shorter healthy life and exacerbates health inequities. Interventions aimed at slowing biological aging may yield dual benefits—improving overall population health and reducing disparities in how long and how well people live.

